# Association between Dietary Pattern and Incidence of Cholesterolemia in Korean Adults: The Korean Genome and Epidemiology Study

**DOI:** 10.3390/nu10010053

**Published:** 2018-01-09

**Authors:** Jieul Lee, Jihye Kim

**Affiliations:** Department of Medical Nutrition, Graduate School of East-West Medical Science, Kyung Hee University, Yongin 17104, Korea; dlwldmf@naver.com

**Keywords:** dietary pattern, Korean Genome and Epidemiology study, low HDL cholesterolemia, high LDL cholesterolemia, prudent pattern, coffee, fat, sweet pattern

## Abstract

We examined the gender-specific association between dietary pattern and risk of developing cholesterolemia based on the data from the Korean Genome and Epidemiology study. A total of 7515 individuals aged 40–69 years participated in this study between 2005 and 2010. Dietary intake was assessed by a semi-quantitative food frequency questionnaire. Low HDL cholesterolemia was defined as a plasma HDL-C level <1.04 mmol/L (men) or <1.30 mmol/L (women), and high LDL cholesterolemia was defined as a plasma LDL-C level >3.37 mmol/L. Multivariate Cox proportional hazard models were used to examine the risk for incident cholesterolemia according to dietary pattern score. Four dietary patterns were derived by gender using factor analysis: prudent pattern; coffee, fat, and sweet pattern; whole grain (men) or white rice and noodle (women) pattern; and westernized pattern. A prudent pattern was inversely associated with risk of low HDL cholesterolemia in both men (Hazard ratio (HR) = 0.76, *p* for trend = 0.0098) and women (HR = 0.78, *p* for trend = 0.0324), whereas the coffee, fat, and sweet pattern was positively associated with risk of high LDL cholesterolemia in men only (HR = 1.26, *p* for trend = 0.0254) after adjustment for potential confounders. Specific dietary patterns were associated with risk of developing cholesterolemia suggesting gender differences.

## 1. Introduction

Dyslipidemia is well recognized as a major cause of cardiovascular disease (CVD) [1,2]. According to the U.S. National Health and Nutrition Examination Survey, the prevalence of dyslipidemia was 53% in U.S. adults [3]. Dyslipidemia is steadily rising in Korea as well as western populations. Data from the Korea National Health and Nutrition Survey reported that the prevalence of dyslipidemia was 57.6% in men and 60.4% in women in 2010 [4].

Dietary pattern is an important determinant of chronic diseases such as type 2 diabetes, hypertension, and metabolic syndrome [5,6,7]. Previous studies have shown the association between dietary pattern and dyslipidemia [7,8,9,10]. A Mediterranean diet characterized by high intake of vegetables, fruits, whole grains, fish, legumes, nuts, and olive oil was positively associated with high-density lipoprotein cholesterol (HDL-C) in a Spanish population aged 40–55 years [8]. In a randomized controlled trial, the Dietary Approaches to Stop Hypertension (DASH) diet, which is high in whole grains, vegetables, fruits, legumes, and low-fat dairy products, reduced low density lipoprotein cholesterol (LDL-C), and total cholesterol compared with a control diet in healthy adults aged >21 years [11]. A study of Northern Chinese adults aged 20–74 years showed that a snack pattern with high intake of biscuits, fried chips, liquid beverages, sweets, and ice cream was positively associated with serum total cholesterol, LDL-C, and triglyceride (TG) [10]. However, the effect of dietary pattern on dyslipidemia has not been confirmed from prospective studies. Furthermore, evidence of the association between dietary pattern and dyslipidemia is very limited for Asian populations.

Dietary patterns vary depending on age, gender, culture, and ethnicity [12]. Koreans, especially older people, consume a very high carbohydrate diet (>70% of energy intake), and such a high carbohydrate intake is associated with changes in blood cholesterol levels among Koreans [13].

In the current study, we investigated the gender-specific association between dietary pattern and risk of developing cholesterolemia (low HDL cholesterolemia or high LDL cholesterolemia) in middle-aged or older Korean adults using data from the Korean Genome and Epidemiology Study (KoGES), which is a large community-based cohort study.

## 2. Materials and Methods

### 2.1. Study Population

This study was based on the KoGES, a large community-based cohort study [14]. The study was conducted to assess the effects of dietary, environmental, and lifestyle determinants on the incidence of chronic diseases in the general Korean population aged 40–69 years living in the Ansan or Ansung areas. Baseline data was collected between 2005 and 2006, and follow-up examinations were performed every 2 years over a 6-year period (2007–2008, 2009–2010). For calculation of follow-up period of participants, data on year, month, and day of follow-up examination were collected. Study participants completed interviewer-administered questionnaires on demographic information and lifestyle factors including dietary habits, their health condition, and medical history, and also anthropometric and biochemical measurements were all conducted biennially.

Among the 7515 subjects who participated at baseline, the following were excluded: 691 participants who refused to participate in the follow-up examination; 3872 participants who had low HDL cholesterolemia at baseline (The participants were excluded to measure incidence of HDL cholesterolemia to predict the risk of low HDL cholesterolemia according to dietary pattern score, which are presented as hazard ratio); 30 participants who had cancer or CVD; 19 participants who had an implausible energy intake (<500 kcal or >6000 kcal); 217 participants who did not complete a food frequency questionnaire (FFQ); and 31 participants who had missing information. Finally, a total of 2655 subjects (1716 men and 939 women) were entered into the low HDL cholesterolemia analysis. Likewise, among the 7515 subjects who participated at baseline, the following were excluded: 691 participants who refused to participate in the follow-up examination; 2395 participants who had high LDL cholesterolemia at baseline (the participants were excluded to measure incidence of LDL cholesterolemia to predict the risk of high LDL cholesterolemia according to dietary pattern score, which are presented as hazard ratio); 69 participants who had cancer or CVD; 26 participants who had an implausible energy intake (<500 kcal or >6000 kcal); 262 participants who did not complete a FFQ; and 67 participants who had missing information. Thus, a total of 4005 subjects (2057 men and 1948 women) were entered into the high LDL cholesterolemia analysis.

The study protocol was approved by the Institutional Review Boards of the Korea Centers for Disease Control and Prevention and Kyung Hee University (KHSIRB-16-022). Written informed consent was obtained from all participants.

### 2.2. Dietary Assessment

Dietary intake was assessed by a validated 106-item, semi-quantitative FFQ [15] at baseline. All study procedures were conducted by trained interviewers. Subjects were asked to report the usual frequency and portion size of each food consumed during the past year. The answer for frequency had nine options for each food: “never/almost,” “once/month,” “2–3 times/month,” “1–2 times/week,” “3–4 times/week,”“5–6 times/week,” “once/day,” “2 times/day,” and “≥3 times/day.” The answer for portion size had three options for each food: “small (1/2 serving/day),” “standard (1 serving/day),” and “large (≥2 servings/day).” The portion size of each food was determined based on the KoGES FFQ guideline. For analysis, food consumption was converted to weekly frequencies and then multiplied by the reported portion sizes.

To identify dietary patterns, food items were categorized into 27 food groups based on the common food groups classified in the Korean Nutrient Database [16]. Because the grain and grain product intakes of Koreans are very high, this group was divided into seven categories: White rice, whole grains, noodles and dumplings, rice cakes, cereal and snacks, breads, and pizza and hamburger. The vegetable group was divided into two categories: Vegetables and Kimchi (traditional fermented, salted vegetables). Kimchi was considered a single vegetable group because it is frequently eaten as a traditional side dish in Korea and contains high levels of sodium. Also, coffee was separated from the beverage group because the consumption of coffee is common between meals or after meals among Korean adults. Daily nutrient intakes were calculated using a food composition table from the Rural Development Administration of Korea [17].

### 2.3. Definition of Low HDL Cholesterolemia and High LDL Cholesterolemia

Plasma levels of HDL-C and LDL-C were measured every 2 years to calculate incidence of low HDL cholesterolemia or high LDL cholesterolemia during the follow-up period. Low HDL cholesterolemia was defined as a plasma HDL-C level <40 mg/dL (1.04 mmol/L) in men or <50 mg/dL (1.30 mmol/L) in women based on the criteria from the joint interim statement of the International Diabetes Federation and the American Heart Association/National Heart, Lung, and Blood Institute [18]. High LDL cholesterolemia was defined as a plasma LDL-C level >130 mg/dL (3.37 mmol/L) based on the criteria of the National Cholesterol Education Program—Adult Treatment Panel III [19].

### 2.4. Other Variables

Demographic factors, socioeconomic status, and lifestyle factors of the subjects at baseline were determined using questionnaires by trained interviewers. This study collected information on education level (≤6 years (elementary school graduate), 7–≤12 years (middle school or high school graduate), and >12 years (college graduate or more)), household income level (<850 US$, 850–<1700 US$, 1700–<2500 US$, and ≥2500 US$), alcohol intake (never, former, and current), and smoking status (never, former, and current). Physical activity level was assessed using the metabolic equivalent of task (MET) hours per day. Subjects reported hours spent on sleep and activities classified according to activity intensity, including sedentary, very light, light, moderate, and heavy activity [20]. Information on anthropometric and biochemical measurements were collected biennially. Height was measured to the nearest 0.1 cm using a stadiometer (Aluminum anthropometer, Samhwa Instrument, Seoul, Korea), and body weight was measured to the nearest 0.1kg while the subject wore light clothes, without shoes, using bioelectrical impedance analysis (Inbody 3.0, Biospace Corp., Seoul, Korea) by trained research staff. Body mass index (BMI) was calculated as weight (kg) divided by height squared (m²). The blood samples were collected after at least eight hours of fasting, and plasma was separated for biochemical testing. The concentrations of TG, total cholesterol, and HDL-C in plasma were measured using an autoanalyzer (ADIVA 1650, Bayer HealthCare, Tarrytown, NY, USA). The concentrations of LDL-C were calculated by the Friedewald equation (total cholesterol—HDL-C—TG/5) [21].

### 2.5. Statistical Analyses

Dietary patterns were separately derived for men and women using factor analysis. Food groups were expressed as the percentage of energy for each food group in order to adjust for weight differences between solid and liquid foods. Food groups were entered into the factor analysis using the FACTOR procedure of SAS software (version 9.4, SAS Institute, Inc., Cary, NC, USA). To identify the number of factors to be retained, we used an eigenvalue >1.3 criterion [22]. The factors were rotated with an orthogonal transformation using a varimax rotation to achieve a simpler structure with greater interpretability [23]. Post-rotated factor loadings revealed that the four factors well described the distinctive dietary patterns in the study population. Factor scores were saved from the principal component analysis for each individual after varimax rotation. Factor scores for each pattern and for each individual were determined by summing the intake of all food group weighted by the factor loading. Factor scores of each dietary pattern were categorized into tertiles for comparison of lifestyle factors and nutrient intake.

Characteristics of subjects at baseline are expressed as number and percentage (categorical variables) or as mean and standard deviation (continuous variables). Comparison of variables at baseline across tertile categories of dietary pattern scores were performed by the Chi-square test and by generalized linear models, as appropriate. Time-dependent Cox proportional hazard models were used to assess hazard ratios (HRs) and 95% confidence intervals (CIs) for developing low HDL cholesterolemia or high LDL cholesterolemia according to tertile category of dietary pattern score. The proportional hazard assumption was confirmed graphically using log-log plots [24] and statistically based on Schoenfeld’s residuals [25] with no major violation of the assumption. In multivariable adjusted models, age was adjusted in model 1. Age, BMI, residential location, education level, household income, smoking status, alcohol intake, and physical activity were adjusted in model 2. All *p* values < 0.05 were considered statistically significant.

## 3. Results

### 3.1. Dietary Patterns in Men and Women

Four dietary patterns were identified for men and women using factor analysis (Table 1). The following four patterns were defined based on the food items that highly loaded: Prudent pattern characterized by high intake of potatoes, legumes, vegetables, mushrooms, fish and shellfish, and seaweed; coffee, fat, and sweet pattern with a higher intake of coffee, oil and fat, and sweets; whole grain pattern (men) or white rice and noodle pattern (women); westernized pattern with high factor loading of cereal and snacks, breads, pizza and hamburgers, nuts, fruits, and dairy products.

### 3.2. Baseline Characteristics of Subjects across Tertiles of Dietary Pattern Score

A follow-up rate of 75% was achieved, resulting in 8928 person-years accrued in the low HDL cholesterolemia analysis and 13,636 person-years accrued in the high LDL cholesterolemia analysis. The average follow-up period was 40.1 months. During a six-year follow-up period, a total of 1073 subjects (602 men and 471 women) developed low HDL cholesterolemia, and 1485 subjects (632 men and 853 women) developed high LDL cholesterolemia.

The characteristics of Korean adults at baseline according to the tertile categories of dietary pattern scores by gender are shown in Table 2 and Table 3. Subjects (both men and women) with higher scores for the prudent pattern were more educated, more likely to live in rural areas, and more likely to have higher income compared to those with low scores for prudent pattern. Subjects (both men and women) with high scores for the coffee, fat, and sweet pattern were younger, more likely to live in rural areas, and more likely to have higher income. Subjects with higher scores for the coffee, fat, and sweet pattern were less likely to have never smoked and more likely to have irregular physical activity in men and were less likely to be a never alcohol drinker in women. Other dietary patterns such as whole grain pattern or white rice and noodle pattern, and westernized pattern were not shown because they were not associated with the risk of cholesterolemia.

### 3.3. Nutrient and Food Intakes across Tertiles of Dietary Pattern Score

Nutrient and food intakes for Korean adults according to the tertile categories of dietary pattern scores by gender are shown in Table 4 and Table 5. In both men and women, subjects with high scores for the prudent pattern have higher intakes of energy and other nutrients, except energy from carbohydrates and food groups such as vegetables, meat and dairy. Subjects with high scores for the coffee, fat, and sweet pattern have lower intakes of energy from protein and carotene regardless of gender.

### 3.4. Association of Dietary Pattern with Low HDL Cholesterolemia or High LDL Cholesterolemia

The hazard ratios (HRs) and 95% confidence intervals (CIs) for the risk of incident low HDL cholesterolemia and high LDL cholesterolemia according to dietary pattern score by gender are shown in Table 6. Subjects with high scores for the prudent pattern had a lower risk of low HDL cholesterolemia in both gender groups. Men in the highest tertile of the prudent pattern had a 26% lower risk of developing low HDL cholesterolemia (HR = 0.74, 95% CI = 0.61–0.91, *p* for trend = 0.0036), and women in the highest tertile of the prudent pattern had a 23% reduced risk of developing low HDL cholesterolemia (HR = 0.77, 95% CI = 0.61–0.96, *p* for trend = 0.0174) compared with those in the lowest tertile of the prudent pattern after adjustment for age. The trend remained after adjustment for potential confounders of age, BMI, residential location, education level, household income, smoking status, alcohol intake, and physical activity in both men (HR = 0.76, 95% CI = 0.62–0.94, *p* for trend = 0.0098) and women (HR = 0.78, 95% CI = 0.62–0.98, *p* for trend = 0.0324). Other dietary patterns were not associated with the risk of incident low HDL cholesterolemia for either men or women. Men in the highest tertile of the coffee, fat, and sweet pattern had a 26% increased risk of developing high LDL cholesterolemia (HR = 1.26, 95% CI = 1.03–1.55, *p* for trend = 0.0254) compared with those in the lowest tertile of the coffee, fat, and sweet pattern after adjustment for potential confounding factors. However, there was no association between the coffee, fat, and sweet pattern and high LDL cholesterolemia in women. Other dietary patterns were not associated with the risk of incident high LDL cholesterolemia for either men or women.

## 4. Discussion

In this large cohort study, specific Korean dietary patterns were associated with the risk of incident dyslipidemia in middle-aged and older Korean adults. Using factor analysis, four dietary patterns were derived from Korean men and women: prudent pattern, coffee, fat, and sweet pattern, whole grain (men) or white rice and noodle (women) pattern, and westernized pattern. During a six-year follow-up period, the prudent pattern characterized by high intakes of potatoes, legumes, vegetables, mushrooms, fish and shellfish, and seaweed was associated with a 22–24% lower risk of developing low HDL cholesterolemia after adjustment for potential confounders in both men and women. In men only, a coffee, fat, and sweet pattern characterized by high intakes of coffee, oil and fat, and sweets was associated with a 26% higher risk of developing high LDL cholesterolemia after adjustment for potential confounding factors. Other dietary patterns were not significantly associated with cholesterolemia in either men or women. These results suggest that a specific dietary pattern might be an independent determinant of cholesterolemia with gender difference in the association between diet and cholesterolemia.

Food composition of the prudent pattern that was inversely associated with low HDL cholesterolemia in this study is similar to the Mediterranean-style diet or DASH diet, which is representative of a healthy dietary pattern, as shown in Western populations. In the Framingham Heart Study, in a seven-year follow-up period, US adults with a higher score regarding the Mediterranean-style pattern had a 32% higher level of HDL-C compared to those with a low pattern score after adjustment for potential confounders [7]. In a cross-sectional study of a Spanish population aged 40–55 years, participants adhering to the Mediterranean dietary pattern had 3.3 mg/dL higher HDL-C levels [8]. The DASH diet was inversely associated with low HDL-C. In a cross-sectional study of Iranian women aged over 30 years, participants in the highest tertile of DASH diet scores were associated with a 78% lower risk of low HDL cholesterolemia compared with those in the lowest tertile of DASH diet scores [26]. Similar to the prudent pattern shown in the present study, in a Greek population aged over 18 years, a dietary pattern with high intakes of fish, vegetables, legumes, cereals, and fruits was positively associated with high HDL-C concentration [6].

There are possible mechanisms to explain an inverse association between a prudent dietary pattern and low HDL cholesterolemia. The prudent dietary pattern includes various foods and food groups that are rich in nutrients and food components such as dietary fiber, antioxidants (beta-carotene, vitamins C and E), polyphenols, dietary fat, and some minerals including calcium, which influence health outcomes. In the present study, participants with higher scores for the prudent pattern had higher intakes of fiber, carotene, vitamin C, and calcium. In a randomized controlled trial of a Swedish population aged 25–65 years, the diet, which was high in dietary fiber, beta-glucan, and polyunsaturated fat for six weeks, was associated with a 5% decrease of HDL-C concentration [27]. Vegetables and legumes, major components of the prudent pattern and contributors to dietary fiber intake, are known to have beneficial effects on blood cholesterol levels [28,29]. Frequent legume consumption (>1 time/day) was associated with a 19% lower risk of low HDL cholesterolemia compared to infrequent legume consumption (<3 times/week) in Iranian adults aged ≥ 19 years [28]. A meta-analysis of 60 controlled trials reported that replacing carbohydrate with fat such as saturated fatty acids, monounsaturated fatty acids, or polyunsaturated fatty acids in diet increased serum HDL cholesterol [30]. Also, omega-3 fatty acids, which are abundant in fish, shellfish, and seaweed, have beneficial effects on serum HDL-C concentration [31,32]. Omega-3 supplementation (2.4 g/day) for eight weeks led to a significant increase in serum HDL level compared with a placebo group in Iranian adolescents aged 10 to 18 years [31]. Calcium intake was inversely associated with elevated total cholesterol and reduced HDL-C level in urban Korean adults aged over 20 years [33]. Altogether, these findings show that the protective effects of the prudent pattern on low HDL cholesterolemia might be due to the combined and synergistic effects of various components, rather than a single nutrient or food component.

It is of interest that the coffee, fat, and sweet pattern was positively associated with high LDL cholesterolemia in men only. Consistent with the present study, a meta-analysis of randomized trials conducted in Western countries revealed positive dose-response relationships between coffee intake and total cholesterol and LDL-C. On average, drinking coffee for 45 days was associated with an increase of 8.1 mg/dL for total cholesterol and 5.4 mg/dL for LDL-C [34]. Coffee oils might be major contributors to the cholesterol-raising effect of coffee. Coffee oils such as cafestol and kahweol may reduce the activity of hepatic LDL receptors and thereby cause extracellular accumulation of LDL-C [35,36]. A previous meta-analysis showed that trials using unfiltered coffee and boiled coffee had a stronger cholesterol-raising effect than did those using filtered coffee because of a higher concentration of coffee oils [34]. The LDL-C raising effect of cafestol might be affected by the apoprotein E polymorphism, and the effect of apoprotein E on the response of cholesterol level to the changes in dietary cafestol differed between men and women [37]. This might partly explain gender differences in the association between coffee, fat, and sweet pattern and the risk of high LDL cholesterolemia as revealed in the present study.

In agreement with our study, a meta-analysis has reported gender differences in the effect of coffee consumption on lipid levels [34]. The underlying mechanism for gender differences on the relationship between dietary factors and disease risk has not been clearly revealed. Gender difference might be associated with sex hormones, sex-specific gene polymorphism, or increased immune inflammatory factors that are related to the regulation of lipid levels [12,38]. Sex hormones such as estrogen lower LDL-C and lipoprotein (a) levels and raise HDL-C level, whereas androgens have an adverse effect on lipid profiles [39]. Also, there are many different pathophysiological and social reasons why the relationship between diet and dyslipidemia may have been only observed in men. Although the present study was unable to specifically determine the reasons, these results propose that gender should be considered in the analysis of association between diet and dyslipidemia, and more research is needed to determine the underlying mechanism.

Older Korean adults commonly drink instant coffee mix or coffee with sugar (sweets) and powered creamer (oil and fat) between meals or after meals [40]. Powdered creamer is composed of palm oil, sweetener, and some additives. Palm oil is well known to have an adverse effect on health—as much as animal fat [41]. In a meta-analysis of clinical trials, palm oil was positively associated with a 0.24 mmol/L increase of LDL-C compared with vegetable oils [42]. Thus, fat sources, perhaps the powered creamer, included in the coffee, fat, and sweet pattern as well as coffee oil might also contribute to the positive association between the coffee, fat, and sweet pattern and high LDL cholesterolemia. Unfortunately, the fat sources including individual fatty acids were not analyzed in this study.

The strength of this study is that, to the best of our knowledge, it is the first to reveal a causal relationship between dietary pattern and the risk of incident dyslipidemia in the Asian population using data from a large scale cohort study with a long period of follow up (~six years). Also, this is a well-established prospective cohort study that used standardized procedures to collect data on exposure and outcomes. However, this study also has certain limitations. Our study population included only middle-aged and older adults, and the findings might not be generalizable to other age groups. Also, the dietary pattern analysis utilized was somewhat subjective regarding decisions on the number of factors extracted and food groupings. In addition, the random measurement error caused by within-person variation in dietary assessment may exist due to the use of the single time-point of FFQ.

## 5. Conclusions

During a six-year follow up period, a prudent dietary pattern characterized by high intake of potatoes, legumes, vegetables, mushrooms, fish and shellfish, and seaweed was associated with a lower risk of developing low HDL cholesterolemia in both Korean men and women. A coffee, fat, and sweet pattern with high intake of coffee, oil and fat, and sweets was associated with a higher risk of developing high LDL cholesterolemia in men only, suggesting gender differences regarding the association between dietary pattern and the risk of cholesterolemia in middle-aged and older Korean adults. In future studies, randomized clinical trials should be conducted to confirm the association between dietary pattern and risks of dyslipidemia and also to reveal the underlying mechanism of this association.

## Figures and Tables

**Table 1:** Factor loading matrix for major factors ascertained from dietary patterns of Korean adults.

Foods or Food Groups	Prudent Pattern (Men/Women)	Coffee, Fat, and Sweet Pattern (Men/Women)	Whole Grain/White Rice and Noodle Pattern (Men/Women)	Westernized Pattern (Men/Women)
White rice	−0.16/−0.12	0.07/−0.08	−0.89/0.89	−0.24/−0.21
Whole grains	−0.08/−0.26	−0.08/0.01	0.94/−0.89	−0.05/−0.19
Noodles and dumpling	−0.12/0.02	−0.07/0.05	−0.21/0.33	0.09/0.05
Rice cakes	0.01/0.15	−0.04/−0.12	0.08/0.02	0.29/0.19
Cereals and snacks	0.08/0.02	0.09/0.05	−0.04/0.16	0.32/0.50
Breads	−0.13/0.11	0.03/0.11	−0.07/0.20	0.48/0.42
Pizza and hamburgers	−0.07/0.01	0.05/0.20	−0.12/0.09	0.37/0.47
Potato and sweet potato	0.32/0.41	−0.13/−0.06	0.14/−0.02	0.11/0.05
Starch jelly	0.14/0.33	0.02/0.02	0.00/0.05	0.00/0.05
Sweets	0.00/−0.05	0.94/0.90	0.02/0.09	−0.01/−0.07
Nuts	0.13/0.18	0.02/0.01	−0.02/−0.03	0.33/0.39
Legumes	0.49/0.51	−0.01/0.00	0.05/−0.10	−0.10/−0.05
Vegetables	0.66/0.61	−0.12/−0.08	−0.02/−0.05	−0.01/−0.13
Kimchi	0.23/0.19	0.05/0.03	0.05/0.08	−0.52/−0.56
Mushrooms	0.50/0.53	0.05/−0.02	0.01/−0.05	0.24/0.18
Fruits	0.18/0.01	0.04/−0.02	0.12/−0.14	0.36/0.39
Meat and its products	0.23/0.34	−0.06/0.05	−0.27/0.15	−0.04/0.07
Eggs	0.09/0.20	0.03/0.13	0.00/−0.03	0.10/0.10
Fish and shellfish	0.53/0.61	−0.02/0.05	−0.05/−0.01	0.24/0.18
Seaweeds	0.50/0.42	0.05/−0.02	0.00/0.01	−0.03/−0.22
Dairy products	0.08/0.10	−0.11/−0.06	0.04/−0.02	0.43/0.26
Soups	0.22/0.37	0.01/−0.03	−0.16/0.07	0.07/0.08
Seasoning	0.37/0.29	0.06/−0.06	0.13/0.02	−0.33/−0.39
Oil and fat	0.00/−0.02	0.88/0.87	−0.01/0.09	0.09/0.06
Coffee	0.00/0.05	0.91/0.86	−0.02/0.09	−0.03/0.02
Carbonated drinks	0.00/−0.03	0.03/0.06	−0.32/0.15	0.23/0.00
Other beverages	0.13/0.14	−0.03/−0.09	−0.11/0.11	0.28/0.26

Food groups with absolute value of factor loading >|0.3|for each dietary pattern are presented in bold.

**Table 2:** Characteristics of Korean adults in low high-density lipoprotein (HDL) cholesterolemia analysis according to the tertile (T) categories of dietary pattern scores.

	Low HDL Cholesterolemia Analysis (*n* = 2655)					
	Prudent Pattern			Coffee, Fat, and Sweet Pattern		
	T1	T2	T3	T1	T2	T3
Men						
No. of participants	572	572	572	572	572	572
Age (year) ^1^	56.1 ± 8.8 ^a^	54.5 ± 8.4 ^b^	56.1 ± 8.7 ^a,^*	57.0 ± 8.7 ^a^	55.0 ± 8.7 ^b^	54.7 ± 8.5 ^b,^***
Residential location (%) ^2^						
Ansan(urban)	58.6	41.1	48.4 ***	64	46.5	37.6 ***
Ansung(rural)	41.4	58.9	51.6	36	53.5	62.4
Education level (%)						
≤6 years	24.8	15.7	17.7 ***	24.8	15.2	18.2 **
7–≤12 years	59.1	60.2	58	56.5	61	59.8
>12 years	16.1	24.1	24.3	18.7	23.8	22
Household income (US$/month) (%)						
<850	32	21.9	25.0 ***	33.6	22.2	23.1 ***
850–<1700	27.1	22	21.2	23.1	26.2	21
1700–<2500	17.7	19.9	15.9	17.1	16.8	19.6
≥2500	23.2	36.2	37.9	26.2	34.8	36.3
Smoking status (%)						
Never	26.1	23.6	25	35.8	22	16.8 ***
Former	40.9	42.5	37.2	37.1	47.4	36.2
Current	33	33.9	37.8	27.1	30.6	47
Alcohol intake (%)						
Never	16.6	13.3	12.9	15	13.3	14.5 *
Former	9.1	6.1	6.5	8.9	4.5	8.2
Current	74.3	80.6	80.6	76.1	82.2	77.3
Physical activity						
MET ^3^, hour/day	25.5 ± 16.7 ^a^	22.6 ± 15.2 ^b^	25.5 ± 14.8 ^a,^*	27.1 ± 16.4 ^a^	24.0 ± 15.1 ^b^	22.4 ± 15.0 ^b,^***
Body mass index (kg/m²)	23.5 ± 2.8	23.8 ± 3.0	23.7 ± 2.7	23.4 ± 2.9 ^a^	23.8 ± 2.7 ^b^	23.8 ± 2.9 ^b,^*
Triglyceride (mmol/L)	1.5 ± 1.6 ^a^	1.4 ± 0.9 ^a,b^	1.3 ± 0.8 ^b,^*	1.4 ± 1.0	1.5 ± 1.5	1.4 ± 0.8
HDL cholesterol (mmol/L)	1.3 ± 0.2	1.3 ± 0.2	1.3 ± 0.2	1.3 ± 0.2 ^a^	1.3 ± 0.2 ^a,b^	1.2 ± 0.2 ^b,^*
LDL ^4^ cholesterol (mmol/L)	3.0 ± 0.8	3.1 ± 0.8	3.1 ± 0.8	2.9 ± 0.8 ^a^	3.1 ± 0.8 ^b^	3.2 ± 0.8 ^c,^***
Women						
No. of participants	313	313	313	313	313	313
Age (year) ^1^	56.6 ± 8.6 ^a^	54.8 ± 8.4 ^b^	53.5 ± 7.8 ^b,^***	57.5 ± 8.3 ^a^	53.0 ± 7.4 ^b^	54.3 ± 8.7 ^b,^***
Residential location (%) ^2^						
Ansan(urban)	59.1	40.6	54.6 ***	62	47.6	44.7 ***
Ansung(rural)	40.9	59.4	45.4	38	52.4	55.3
Education level (%)						
≤6 years	50.2	35.8	27.2 ***	50.8	28.4	33.9 ***
7–≤12 years	45	55.3	64.2	43.8	65.5	55.3
>12 years	4.8	8.9	8.6	5.4	6.1	10.8
Household income (US$/month) (%)						
<850	46.3	31.3	26.8 ***	42.2	29.7	32.6 ***
850–<1700	24.3	20.1	26.2	28.1	22.4	20.1
1700–<2500	13.1	20.5	17.6	9.9	19.8	21.4
≥2500	16.3	28.1	29.4	19.8	28.1	25.9
Smoking status (%)						
Never	97.1	98.1	97.8	98.4	98.1	96.5
Former	1	1	0.6	0.6	0.3	1.6
Current	1.9	0.9	1.6	1	1.6	1.9
Alcohol intake (%)						
Never	68.7	61	64.9	76.4	58.5	59.7 ***
Former	1.6	2.3	0.3	1.3	1.9	1
Current	29.7	36.7	34.8	22.3	39.6	39.3
Physical activity						
MET ^3^, hour/day	22.9 ± 15.5	23.2 ± 14.3	22.9 ± 15.8	24.8 ± 16.7 ^a^	21.0 ± 13.9 ^b^	23.1 ± 14.7 ^a,b,^*
Body mass index (kg/m²)	24.3 ± 3.3	24.5 ± 3.3	23.9 ± 3.3	23.9 ± 3.5	24.6 ± 3.4	24.1 ± 3.0
Triglyceride (mmol/L)	1.1 ± 0.5	1.1 ± 0.5	1.0 ± 0.5	1.2 ± 0.6 ^a^	1.1 ± 0.4 ^a,b^	1.0 ± 0.4 ^b,^*
HDL cholesterol(mmol/L)	1.5 ± 0.2	1.5 ± 0.2	1.5 ± 0.2	1.5 ± 0.2	1.5 ± 0.2	1.5 ± 0.2
LDL ^4^ cholesterol(mmol/L)	3.2 ± 0.8	3.3 ± 0.8	3.3 ± 0.7	3.3 ± 0.8	3.3 ± 0.7	3.3 ± 0.8

^1^ Continuous variables are summarized as mean ± standard deviation and compared using the generalized linear model; ^2^ Categorized variables are summarized as percentages and compared using Chi-square test; ^3^ MET: metabolic equivalent of task; ^4^ LDL: low-density lipoprotein; ^a,b,c^ Difference of variables among tertile groups were examined by Post-hoc test (Tukey’s test for multiple comparisons). The unlike superscripts mean significant differences across tertile groups. * *p* for trend < 0.05; ** *p* for trend < 0.001; *** *p* for trend < 0.0001.

**Table 3:** Characteristics of Korean adults in high low-density lipoprotein (LDL) cholesterolemia analysis according to the tertile (T) categories of dietary pattern scores.

	High LDL Cholesterolemia Analysis (*n* = 4005)					
	Prudent Pattern			Coffee, Fat, and Sweet Pattern		
	T1	T2	T3	T1	T2	T3
Men						
No. of participants	685	686	686	685	686	686
Age (year) ^1^	56.6 ± 8.9	55.5 ± 8.8	55.9 ± 8.5	57.3 ± 8.9 ^a^	55.3 ± 8.5 ^b^	55.4 ± 8.7 ^b,^***
Residential location (%) ^2^						
Ansan(urban)	69.2	50	52.8 ***	72.1	55.5	44.3 ***
Ansung(rural)	30.8	50	47.2	27.9	44.5	55.7
Education level (%)						
≤6 years	27.9	19.2	16.6 ***	26.9	15.7	21.1 ***
7–≤12 years	57.4	60.5	60.1	56.9	61.8	59.2
>12 years	14.7	20.3	23.3	16.2	22.5	19.7
Household income (US$/month) (%)						
<850	33.4	24.8	24.6 ***	34.6	22	26.2 ***
850–<1700	30.1	24.3	24.1	27.5	28	23
1700–<2500	16.2	17.8	15.7	15.9	15.7	18.1
≥2500	20.3	33.1	35.6	22	34.3	32.7
Smoking status (%)						
Never	24.8	26.7	24.1	34.6	24.5	16.5 ***
Former	38.4	35.6	35.7	34.6	40.7	34.4
Current	36.8	37.8	40.2	30.8	34.8	49.1
Alcohol intake (%)						
Never	19.1	19.1	16.5 *	19.4	16.3	19.0 *
Former	10.4	5.5	8.9	10.5	7.2	7.1
Current	70.5	75.4	74.6	70.1	76.5	73.9
Physical activity						
MET ^3^, hour/day	26.5 ± 16.7 ^a^	24.2 ± 16.0 ^b^	25.6 ± 15.9 ^a,b,^*	27.6 ± 17.1 ^a^	25.1 ± 15.5 ^b^	23.6 ± 15.7 ^b,^***
Body mass index (kg/m²)	24.0 ± 3.0	24.2 ± 3.0	24.1 ± 2.9	23.8 ± 3.0	24.4 ± 2.9	24.1 ± 3.0
Triglyceride (mmol/L)	1.9 ± 1.9	1.9 ± 1.9	1.7 ± 1.3 *	1.9 ± 1.5	2.0 ± 2.0	1.8 ± 1.6
HDL^4^ cholesterol (mmol/L)	1.1 ± 0.3 ^a^	1.1 ± 0.3 ^a,b^	1.1 ± 0.3 ^b,^*	1.1 ± 0.3	1.1 ± 0.3	1.1 ± 0.3
LDL cholesterol (mmol/L)	2.5 ± 0.7	2.5 ± 0.7	2.6 ± 0.6	2.5 ± 0.7	2.5 ± 0.7	2.6 ± 0.6
Women						
No. of participants	649	650	649	649	650	649
Age (year) ^1^	57.9 ± 9.2 ^a^	55.5 ± 9.0 ^b^	55.4 ± 8.7 ^b,^***	58.1 ± 8.7 ^a^	54.7 ± 8.8 ^b^	56.2 ± 9.4 ^c,^***
Residential location (%) ^2^						
Ansan(urban)	71	49.9	65.2 ***	70.1	59.5	59.5 ***
Ansung(rural)	29	50.1	34.8	29.9	40.5	40.5
Education level (%)						
≤6 years	57.8	41.1	39.2 ***	53.6	39.2	45.1 ***
7–≤12 years	39.4	50.6	53.9	43.8	53.2	47
>12 years	2.8	8.3	6.9	2.6	7.6	7.9
Household income (US$/month) (%)						
<850	53.3	38.5	41.3 ***	50.8	38	44.2 ***
850–<1700	23.4	20.3	24.4	24.2	25.2	18.6
1700–<2500	11.6	19.4	14.9	12.5	16.5	17
≥2500	11.7	21.8	19.4	12.5	20.3	20.2
Smoking status (%)						
Never	97.5	97.4	96.9	98.1	97.7	96
Former	0.8	0.1	0.3	0.2	0.2	0.9
Current	1.7	2.5	2.8	1.7	2.1	3.1
Alcohol intake (%)						
Never	73.7	68	72.9	82.4	68.2	63.9 ***
Former	1.7	1.9	1.7	1.2	2	2
Current	24.6	30.1	25.4	16.3	29.8	34.1
Physical activity						
MET ^3^, hour/day	24.0 ± 16.2	23.0 ± 14.8	23.3 ± 16.0	24.3 ± 16.2	23.3 ± 15.2	22.8 ± 15.7
Body mass index (kg/m²)	24.6 ± 3.3	24.5 ± 3.1	24.6 ± 3.3	24.2 ± 3.3 ^a^	25.0 ± 3.2 ^b^	24.6 ± 3.1 ^a,^***
Triglyceride (mmol/L)	1.5 ± 1.0	1.5 ± 1.0	1.5 ± 1.0	1.6 ± 1.2 ^a^	1.5 ± 0.9 ^a,b^	1.4 ± 0.9 ^b,^*
HDL ^4^ cholesterol (mmol/L)	1.1 ± 0.2 ^a^	1.1 ± 0.3 ^a,b^	1.2 ± 0.3 ^b,^*	1.1 ± 0.2 ^a^	1.2 ± 0.3 ^b^	1.2 ± 0.3 ^b,^**
LDL cholesterol (mmol/L)	2.7 ± 0.5	2.7 ± 0.5	2.7 ± 0.5	2.7 ± 0.5	2.7 ± 0.4	2.7 ± 0.5 *

^1^ Continuous variables are summarized as mean ± standard deviation and compared using the generalized linear model; ^2^ categorized variables are summarized as percentages and compared using Chi-square test; ^3^ MET: metabolic equivalent of task; ^4^ HDL: high-density lipoprotein; ^a,b,c^ difference of variables among tertile groups were examined by post-hoc test (Tukey’s test for multiple comparisons). The unlike superscripts mean significant differences across tertile groups. * *p* for trend < 0.05; ** *p* for trend < 0.001; *** *p* for trend < 0.0001.

**Table 4:** Nutrient and food intakes of Korean in low high-density lipoprotein cholesterolemia analysis according to the tertile (T) categories of dietary pattern scores (*n* = 2655) ^1^.

	Prudent Pattern			Coffee, Fat, and Sweet Pattern		
	T1	T2	T3	T1	T2	T3
Men						
No. of participants (1716)	572	572	572	572	572	572
Energy (kcal/day)	1901.4 ± 558.2 ^a^	2081.2 ± 571.8 ^b^	2146.2 ± 653.9 ^b,^***	2031.0 ± 656.3 ^a^	2172.9 ± 563.7 ^b^	1924.9 ± 564.7 ^c,^***
Percentage from energy						
Carbohydrate (%)	76.2 ± 5.6 ^a^	72.0 ± 6.0 ^b^	68.3 ± 6.6 ^c,^***	72.4 ± 8.0	71.5 ± 6.2	72.5 ± 6.2 *
Protein (%)	11.7 ± 1.4 ^a^	13.1 ± 1.6 ^b^	14.9 ± 2.2 ^c,^***	13.5 ± 2.5 ^a^	13.4 ± 1.9 ^a^	12.8 ± 1.9 ^b,^***
Fat (%)	12.2 ± 4.5 ^a^	14.8 ± 4.8 ^b^	16.9 ± 5.0 ^c,^***	14.1 ± 5.9 ^a^	15.1 ± 4.7 ^b^	14.8 ± 4.7 ^a,b,^*
Fiber (g/day)	4.5 ± 1.9 ^a^	6.0 ± 2.1 ^b^	7.3 ± 3.2 ^c,^***	6.0 ± 3.2 ^a^	6.2 ± 2.5 ^a^	5.5 ± 2.3 ^b,^***
Vitamin A (μg RE ^2^/day)	309.3 ± 193.2 ^a^	468.1 ± 235.3 ^b^	668.3 ± 415.9 ^c,^***	504.3 ± 422.5 ^a^	507.1 ± 273.5 ^a^	434.2 ± 271.6 ^b,^**
Carotene (μg/day)	1517.2 ± 1024.6 ^a^	2344.3 ± 1337.4 ^b^	3430.8 ± 2340.6 ^c,^***	2556.4 ± 2346.0 ^a^	2533.7 ± 1515.1 ^a^	2202.2 ± 1511.4 ^b,^*
Vitamin C (mg/day)	65.4 ± 38.3 ^a^	99.9 ± 48.4 ^b^	127.7 ± 67.5 ^c,^***	97.1 ± 68.2 ^a,b^	104.3 ± 54.2 ^a^	91.7 ± 51.4 ^b,^*
Calcium (mg/day)	303.8 ± 175.9 ^a^	424.7 ± 191.2 ^b^	540.7 ± 269.0 ^c,^***	425.2 ± 286.0 ^a,b^	450.1 ± 214.9 ^a^	394.0 ± 196.1 ^b,^***
Sodium (mg/day)	2187.5 ± 1171.9 ^a^	2932.5 ± 1302.3 ^b^	3618.1 ± 1789.5 ^c,^***	2934.0 ± 1797.4	2985.9 ± 1423.4	2818.3 ± 1423.4
Cholesterol (mg/day)	111.9 ± 90.9 ^a^	161.7 ± 99.2 ^b^	209.8 ± 125.5 ^c,^***	158.7 ± 123.9 ^a,b^	175.0 ± 104.2 ^a^	149.6 ± 110.0 ^b,^**
Food intake (servings/day)						
Grains	4.6 ± 1.8 ^a^	4.3 ± 1.5 ^b^	4.0 ± 1.4 ^c,^***	4.3 ± 1.6 ^a^	4.6 ± 1.6 ^b^	4.0 ± 1.5 ^c,^***
Vegetables	5.0 ± 2.9 ^a^	7.6 ± 3.4 ^b^	10.6 ± 5.8 ^c,^***	8.0 ± 6.0 ^a^	8.0 ± 4.1 ^a^	7.3 ± 4.1 ^b,^*
Fruit	2.7 ± 2.5 ^a^	4.0 ± 3.0 ^b^	4.3 ± 3.1 ^b,^***	3.4 ± 2.9 ^a^	4.1 ± 3.2 ^b^	3.5 ± 2.6 ^a,^**
Meat	0.3 ± 0.3 ^a^	0.5 ± 0.5 ^b^	0.6 ± 0.6 ^c,^***	0.5 ± 0.6 ^a^	0.5 ± 0.4 ^a^	0.4 ± 0.4 ^b,^*
Dairy	0.5 ± 0.8 ^a^	0.7 ± 0.8 ^b^	0.7 ± 0.9 ^b,^**	0.7 ± 1.0 ^a^	0.7 ± 0.9 ^a^	0.5 ± 0.6 ^b,^***
Women						
No. of participants (939)	313	313	313	313	313	313
Energy (kcal/day)	1782.6 ± 557.1 ^a^	1908.8 ± 545.8 ^a^	2074.5 ± 811.3 ^b,^***	1820.8 ± 643.1 ^a^	2116.8 ± 724.4 ^b^	1828.4 ± 560.4 ^a,^***
Percentage from energy						
Carbohydrate (%)	78.5 ± 4.9 ^a^	73.8 ± 5.2 ^b^	68.6 ± 7.2 ^c,^***	75.4 ± 7.2 ^a^	72.3 ± 7.0 ^b^	73.1 ± 6.8 ^b,^***
Protein (%)	11.4 ± 1.4 ^a^	12.9 ± 1.4 ^b^	15.1 ± 2.5 ^c,^***	12.9 ± 2.5 ^a^	13.6 ± 2.4 ^b^	13.0 ± 2.2 ^a,^**
Fat (%)	10.2 ± 4.0 ^a^	13.3 ± 4.2 ^b^	16.3 ± 5.4 ^c,^***	11.6 ± 5.1 ^a^	14.1 ± 5.1 ^b^	14.0 ± 5.1 ^b,^***
Fiber (g/day)	4.7 ± 1.9 ^a^	5.7 ± 2.1 ^b^	7.6 ± 4.0 ^c,^***	5.8 ± 3.1 ^a^	6.6 ± 3.2 ^b^	5.6 ± 2.8 ^a,^***
Vitamin A (μg RE ^2^/day)	298.2 ± 195.9 ^a^	438.3 ± 230.0 ^b^	727.9 ± 561.0 ^c,^***	461.5 ± 408.8 ^a^	556.8 ± 467.0 ^b^	446.1 ± 331.5 ^a,^*
Carotene (μg/day)	1491.4 ± 1059.6 ^a^	2192.2 ± 1281.3 ^b^	3775.0 ± 3137.7 ^c,^***	2410.3 ± 2315.8 ^a,b^	2793.7 ± 2524.8 ^a^	2254.7 ± 1861.0 ^b,^*
Vitamin C (mg/day)	77.5 ± 46.2 ^a^	106.2 ± 56.1 ^b^	144.4 ± 78.5 ^c,^***	102.0 ± 70.5 ^a^	126.0 ± 74.7 ^b^	100.2 ± 52.3 ^a,^***
Calcium (mg/day)	318.1 ± 193.0 ^a^	432.0 ± 188.9 ^b^	615.5 ± 335.7 ^c,^***	419.2 ± 274.6 ^a^	523.6 ± 316.4 ^b^	422.9 ± 219.5 ^a,^***
Sodium (mg/day)	1980.0 ± 1068.0 ^a^	2497.5 ± 1214.7 ^b^	3327.2 ± 1879.9 ^c,^***	2429.8 ± 1489.3 ^a^	2828.4 ± 1608.4 ^b^	2546.5 ± 1479.1 ^a,b,^*
Cholesterol (mg/day)	83.7 ± 62.5 ^a^	139.4 ± 98.3 ^b^	217.4 ± 171.0 ^c,^***	119.8 ± 104.1 ^a^	176.0 ± 159.8 ^b^	144.7 ± 117.9 ^a,^***
Food intake (servings/day)						
Grains	3.9 ± 1.5	3.7 ± 1.3	3.8 ± 1.7	3.7 ± 1.4 ^a^	4.1 ± 1.8 ^b^	3.6 ± 1.2 ^a,^***
Vegetables	5.0 ± 2.6 ^a^	7.1 ± 3.4 ^b^	11.1 ± 6.8 ^c,^***	7.5 ± 5.5 ^a^	8.5 ± 5.8 ^b^	7.2 ± 4.3 ^a,^*
Fruit	4.4 ± 3.9 ^a^	5.3 ± 3.6 ^b^	5.4 ± 4.0 ^b,^*	4.5 ± 3.9 ^a^	5.9 ± 3.7 ^b^	4.9 ± 3.6 ^a,^**
Meat	0.2 ± 0.2 ^a^	0.3 ± 0.2 ^b^	0.5 ± 0.7 ^c,^***	0.3 ± 0.4 ^a^	0.4 ± 0.5 ^b^	0.3 ± 0.4 ^a,b,^*
Dairy	0.7 ± 0.9 ^a^	0.9 ± 0.9 ^b^	1.0 ± 1.0 ^b,^***	0.8 ± 0.9 ^a^	1.0 ± 1.1 ^b^	0.7 ± 0.8 ^a,^***

^1^ Continuous variables are summarized as mean ± standard deviation and compared using the generalized linear model; ^2^ RE: retinol equivalent; ^a,b,c^ difference of variables among tertile groups were examined by post-hoc test (Tukey’s test for multiple comparisons). The unlike superscripts mean significant differences across tertile groups. * *p* for trend < 0.05; ** *p* for trend < 0.001; *** *p* for trend < 0.0001.

**Table 5:** Nutrient and food intakes of Korean in high low-density lipoprotein cholesterolemia analysis according to the tertile (T) categories of dietary pattern scores (*n* = 4005) ^1^.

	Prudent Pattern			Coffee, Fat, and Sweet Pattern		
	T1	T2	T3	T1	T2	T3
Men						
No. of participants (2057)	685	686	686	685	686	686
Energy (kcal/day)	1835.6 ± 540.5 ^a^	2003.4 ± 569.7 ^b^	2163.3 ± 700.4 ^c,^***	1974.1 ± 704.0 ^a^	2138.5 ± 575.3 ^b^	1890.0 ± 550.3 ^c,^***
Percentage from energy						
Carbohydrate (%)	76.8 ± 5.7 ^a^	72.9 ± 6.0 ^b^	68.9 ± 6.6 ^c,^***	73.5 ± 7.9 ^a^	72.0 ± 6.5 ^b^	73.1 ± 6.1 ^a,^**
Protein (%)	11.5 ± 1.4 ^a^	13.0 ± 1.6 ^b^	14.8 ± 2.2 ^c,^***	13.2 ± 2.5 ^a^	13.4 ± 2.1 ^a^	12.7 ± 2.0 ^b,^***
Fat (%)	11.7 ± 4.7 ^a^	14.1 ± 4.8 ^b^	16.3 ± 4.9 ^c,^***	13.3 ± 5.9 ^a^	14.6 ± 4.9 ^b^	14.2 ± 4.6 ^b,^***
Fiber (g/day)	4.3 ± 1.9 ^a^	5.8 ± 2.1 ^b^	7.4 ± 3.3 ^c,^***	5.9 ± 3.4 ^a^	6.2 ± 2.6 ^a^	5.5 ± 2.2 ^b,^***
Vitamin A (μg RE ^2^/day)	289.7 ± 189.4 ^a^	453.6 ± 240.3 ^b^	671.4 ± 429.3 ^c,^***	482.8 ± 424.2 ^a^	501.4 ± 304.6 ^b^	430.8 ± 276.0 ^a,^**
Carotene (μg/day)	1441.1 ± 1006.5 ^a^	2297.5 ± 1380.8 ^b^	3435.1 ± 2420.4 ^c,^***	2456.0 ± 2355.9 ^a^	2518.6 ± 1678.6 ^a^	2199.6 ± 1536.9 ^b,^*
Vitamin C (mg/day)	61.1 ± 38.4 ^a^	95.5 ± 47.3 ^b^	130.9 ± 69.4 ^c,^***	94.1 ± 71.7 ^a^	101.9 ± 57.2 ^b^	91.5 ± 50.0 ^a,^*
Calcium (mg/day)	273.4 ± 157.8 ^a^	403.2 ± 183.4 ^b^	551.4 ± 289.8 ^c,^***	408.1 ± 302.5 ^a,b^	435.0 ± 221.4 ^a^	385.0 ± 198.9 ^b,^**
Sodium (mg/day)	2080.1 ± 1203.8 ^a^	2867.8 ± 1310.0 ^b^	3579.2 ± 1820.2 ^c,^***	2810.2 ± 1864.8	2935.1 ± 1509.6	2782.9 ± 1355.4
Cholesterol (mg/day)	99.5 ± 85.7 ^a^	147.6 ± 93.6 ^b^	212.7 ± 134.4 ^c,^***	148.8 ± 128.6 ^a^	170.6 ± 113.8 ^b^	140.5 ± 103.3 ^a,^***
Food intake (servings/day)						
Grains	4.4 ± 1.6 ^a^	4.2 ± 1.5 ^b^	4.1 ± 1.6 ^b,^*	4.3 ± 1.7 ^a^	4.5 ± 1.5 ^b^	3.9 ± 1.4 ^c,^***
Vegetables	4.7 ± 3.0 ^a^	7.4 ± 3.6 ^b^	10.6 ± 5.9 ^c,^***	7.6 ± 6.1	7.9 ± 4.5	7.2 ± 4.0
Fruit	2.5 ± 2.6 ^a^	3.8 ± 3.0 ^b^	4.3 ± 3.5 ^c,^***	3.2 ± 3.3 ^a^	3.9 ± 3.3 ^b^	3.5 ± 2.7 ^a,^**
Meat	0.3 ± 0.3 ^a^	0.4 ± 0.5 ^b^	0.6 ± 0.5 ^c,^***	0.4 ± 0.6 ^a^	0.5 ± 0.5 ^b^	0.4 ± 0.4 ^c,^***
Dairy	0.4 ± 0.7 ^a^	0.6 ± 0.8 ^b^	0.7 ± 0.9 ^c,^***	0.6 ± 1.0 ^a^	0.7 ± 0.8 ^a^	0.5 ± 0.7 ^b,^**
Women						
No. of participants (1948)	649	650	649	649	650	649
Energy (kcal/day)	1678.6 ± 523.8 ^a^	1882.3 ± 544.5 ^b^	1975.7 ± 760.8 ^c,^***	1760.1 ± 636.0 ^a^	2049.8 ± 713.5 ^b^	1726.5 ± 467.5 ^a,^***
Percentage from energy						
Carbohydrate (%)	79.9 ± 5.2 ^a^	74.9 ± 5.3 ^b^	70.4 ± 7.4 ^c,^***	77.2 ± 7.2 ^a^	73.4 ± 7.3 ^b^	74.7 ± 6.4 ^c,^***
Protein (%)	11.1 ± 1.4 ^a^	12.8 ± 1.5 ^b^	14.8 ± 2.5 ^c,^***	12.5 ± 2.3 ^a^	13.5 ± 2.6 ^b^	12.7 ± 2.1 ^a,^***
Fat (%)	9.0 ± 4.1 ^a^	12.4 ± 4.2 ^b^	14.8 ± 5.5 ^c,^***	10.3 ± 5.2 ^a^	13.1 ± 5.3 ^b^	12.6 ± 4.8 ^b,^***
Fiber (g/day)	4.4 ± 1.9 ^a^	5.7 ± 2.1 ^b^	7.3 ± 3.6 ^c,^***	5.3 ± 2.6 ^a^	6.6 ± 3.3 ^b^	5.5 ± 2.5 ^a,^***
Vitamin A (μg RE ^2^/day)	261.1 ± 179.5 ^a^	435.9 ± 239.0 ^b^	684.3 ± 512.6 ^c,^***	390.4 ± 315.7 ^a^	552.9 ± 461.9 ^b^	437.8 ± 339.9 ^a,^***
Carotene (μg/day)	1351.4 ± 993.3 ^a^	2212.0 ± 1337.1 ^b^	3572.4 ± 2869.5 ^c,^***	2012.9 ± 1709.2 ^a^	2847.8 ± 2555.3 ^b^	2274.2 ± 1922.6 ^a,^***
Vitamin C (mg/day)	71.0 ± 45.7 ^a^	102.4 ± 53.4 ^b^	134.8 ± 76.1 ^c,^***	90.6 ± 60.3 ^a^	121.9 ± 76.6 ^b^	95.6 ± 51.9 ^a,^***
Calcium (mg/day)	270.2 ± 157.7 ^a^	424.9 ± 202.3 ^b^	572.1 ± 328.5 ^c,^***	371.2 ± 258.9 ^a^	493.1 ± 308.6 ^b^	402.8 ± 221.5 ^a,^***
Sodium (mg/day)	1889.6 ± 1137.2 ^a^	2561.1 ± 1205.8 ^b^	3312.6 ± 1879.3 ^c,^***	2269.5 ± 1353.7 ^a^	2889.8 ± 1723.7 ^b^	2603.5 ± 1514.8 ^c,^***
Cholesterol (mg/day)	64.5 ± 55.2 ^a^	126.5 ± 79.4 ^b^	199.2 ± 175.6 ^c,^***	110.5 ± 119.8 ^a^	162.4 ± 156.7 ^b^	117.2 ± 93.5 ^a,^***
Food intake (servings/day)						
Grains	3.8 ± 1.3	3.8 ± 1.3	3.7 ± 1.5	3.7 ± 1.3 ^a^	4.1 ± 1.6 ^b^	3.5 ± 1.2 ^c,^***
Vegetables	4.6 ± 2.7 ^a^	7.1 ± 3.3 ^b^	11.0 ± 7.0 ^c,^***	6.7 ± 4.6 ^a^	8.8 ± 6.5 ^b^	7.2 ± 4.7 ^a,^***
Fruit	4.1 ± 3.9 ^a^	5.0 ± 3.8 ^b^	4.9 ± 3.7 ^b,^***	4.3 ± 4.0 ^a^	5.5 ± 4.4 ^b^	4.2 ± 2.7 ^a,^***
Meat	0.1 ± 0.2 ^a^	0.2 ± 0.2 ^b^	0.4 ± 0.8 ^c,^***	0.2 ± 0.6 ^a^	0.3 ± 0.5 ^b^	0.2 ± 0.2 ^a,^**
Dairy	0.5 ± 0.7 ^a^	0.8 ± 1.0 ^b^	0.9 ± 1.1 ^b,^***	0.7 ± 1.0 ^a^	0.9 ± 1.0 ^b^	0.7 ± 0.8 ^a,^**

^1^ Continuous variables are summarized as mean ± SD and compared using the generalized linear model; ^2^ RE: retinol equivalent; ^a, b, c^ difference of variables among tertile groups were examined by post-hoc test (Tukey’s test for multiple comparisons). The unlike superscripts mean significant differences across tertile groups. * *p* for trend < 0.05; ** *p* for trend < 0.001; *** *p* for trend < 0.0001.

**Table 6:** Hazard ratios (HRs) and 95% confidence intervals (CIs) for the risk of incident cholesterolemia according to dietary pattern ^1^.

	T1	T2	T3	*p* for Trend
**Low HDL ^2^ Cholesterolemia Analysis (*n* = 2655)**				
Prudent pattern				
*Men*				
No. of participants (No. of cases)	572 (219)	572 (208)	572 (175)	
Model 1	1.00	0.97 (0.80–1.17)	0.74 (0.61–0.91)	0.0036
Model 2	1.00	0.99 (0.81–1.20)	0.76 (0.62–0.94)	0.0098
*Women*				
No. of participants (No. of cases)	313 (179)	313 (145)	313 (147)	
Model 1	1.00	0.80 (0.64–1.00)	0.77 (0.61–0.96)	0.0174
Model 2	1.00	0.80 (0.64–1.00)	0.78 (0.62–0.98)	0.0324
Coffee, fat, and sweet pattern				
*Men*				
No. of participants (No. of cases)	572 (189)	572 (203)	572 (210)	
Model 1	1.00	1.09 (0.89–1.33)	1.10 (0.90–1.34)	0.3507
Model 2	1.00	1.06 (0.87–1.30)	1.02 (0.83–1.26)	0.8364
*Women*				
No. of participants (No. of cases)	313 (160)	313 (156)	313 (155)	
Model 1	1.00	1.06 (0.84–1.33)	1.12 (0.90–1.40)	0.3197
Model 2	1.00	1.01 (0.80–1.27)	1.15 (0.91–1.44)	0.2370
**High LDL ^3^ Cholesterolemia Analysis (*n* = 4005)**				
Prudent pattern				
*Men*				
No. of participants (No. of cases)	685 (214)	686 (213)	686 (205)	
Model 1	1.00	1.03 (0.85–1.24)	0.97 (0.80–1.18)	0.7678
Model 2	1.00	0.95 (0.78–1.15)	0.90 (0.74–1.10)	0.3124
*Women*				
No. of participants (No. of cases)	649 (285)	650 (299)	649 (269)	
Model 1	1.00	1.02 (0.87–1.20)	0.90 (0.76–1.07)	0.2175
Model 2	1.00	0.97 (0.82–1.15)	0.88 (0.75–1.05)	0.1512
Coffee, fat, and sweet pattern				
*Men*				
No. of participants (No. of cases)	685 (180)	686 (210)	686 (242)	
Model 1	1.00	1.14 (0.94–1.40)	1.36 (1.12–1.65)	0.0017
Model 2	1.00	1.08 (0.88–1.33)	1.26 (1.03–1.55)	0.0254
*Women*				
No. of participants (No. of cases)	649 (283)	650 (284)	649 (286)	
Model 1	1.00	0.98 (0.83–1.16)	0.99 (0.84–1.16)	0.8771
Model 2	1.00	0.97 (0.82–1.15)	0.97 (0.82–1.14)	0.6913

^1^ Calculated using the Cox proportional hazard model; Model 1 adjusted for age; Model 2 adjusted for age, body mass index, residential location, education level, household income, smoking status, alcohol intake and physical activity. ^2^ HDL: High-density lipoprotein; ^3^ LDL: low-density lipoprotein.
